# Influence of the number of washings for embryos on non-invasive preimplantation chromosome screening results

**DOI:** 10.3389/fendo.2024.1363851

**Published:** 2024-03-26

**Authors:** Xiaomei Kang, Meiting Wen, Jie Zheng, Fangxin Peng, Ni Zeng, Zhu Chen, Yanting Wu, Hong Sun

**Affiliations:** ^1^ Reproductive Medicine, Maternal and Child Health Hospital of Hubei Province, Wuhan, Hubei, China; ^2^ Department of Obstetrics and Gynecology, The First People’s Hospital of Zigong, Zigong, China

**Keywords:** non-invasive chromosome screening, free chromosome, embryo, assisted reproductive technology (ART), embryo washing

## Abstract

**Objective:**

To explore the effect of varying numbers of embryo washings prior to blastocyst formation in non-invasive preimplantation chromosome screening (NICS) on the accuracy of NICS results.

**Methods:**

In this study, 68 blastocysts from preimplantation genetic testing (PGT)-assisted pregnancy were collected at our institution. On the fourth day of embryo culture, the embryos were transferred to a new medium for blastocyst culture and were washed either three times (NICS1 group) or ten times (NICS2 group). A trophectoderm (TE) biopsy was performed on the blastocysts, and the corresponding embryo culture media were collected for whole genome amplification (WGA) and high-throughput sequencing.

**Results:**

The success rate of WGA was 100% (TE biopsy), 76.7% (NICS1 group), and 89.5% (NICS2 group). The success rate of WGA in embryo medium on days 5 and 6 of culture was 75.0% (33/44) and 100% (24/24), respectively. Using TE as the gold standard, the karyotype concordance rate between the results of the NICS1 and NICS2 groups’ embryo culture medium samples and TE results was 43.5% (10/23) and 73.5% (25/34), respectively. The sensitivity and specificity of detecting chromosomal abnormalities were higher in the NICS2 group than in the NICS1 group when TE was used (83.3% vs 60.0%; 62.5% vs 30.8%, respectively). The false-positive rate and false-negative rate (i.e., misdiagnosis rate and missed diagnosis rate, respectively) were lower in the NICS2 group than in the NICS1 group (37.5% vs 69.2%; 16.7% vs 40.0%, respectively).

**Conclusion:**

The NICS yielded favorable results after ten washings of the embryos. These findings provide a novel method for lowering the amount of cell-free DNA contamination from non-embryonic sources in the medium used for embryo development, optimizing the sampling procedure and improving the accuracy of the NICS test.

## 1 Introduction

Chromosomal abnormalities in embryos are one of the main causes of pregnancy failure in *in vitro* fertilization (IVF). The clinical application of preimplantation genetic testing (PGT) allows for the selection and transfer of chromosomally normal high-quality embryos for transplantation, considerably increasing the embryo implantation rate and clinical pregnancy rate and reducing the miscarriage rate (1). The clinical application value of PGT is affirmative. However, due to the invasiveness of PGT and the lack of long-term safety assessment of progeny (2, 3), the clinical use of PGT is currently limited to certain populations, with PGT not being frequently employed in clinical settings.

In-depth research on embryo culture media has revealed that embryonic genomics can be studied from the culture medium (4, 5), leading to the development of non-invasive chromosome screening technology (NICS). NICS screens embryo chromosomal conditions by detecting free genetic material in the embryo culture media, allowing the selection and transfer of chromosomally normal high-quality embryos, thereby improving pregnancy outcomes and reducing the occurrence of fetal birth defects. This optimization of embryo selection provides personalized guidance for embryo transfer, ultimately enhancing the success rate of assisted reproductive technology.

The existence of cell-free DNA (cfDNA) in the embryo culture media is a key factor in NICS detection. Throughout the embryo culture process, the cfDNA in the culture medium is continuously produced and degraded. Previous studies have shown that cfDNA originates from the blastocyst, cumulus cells, and polar bodies (6–9). The presence of non-embryonic cfDNA in the culture media introduces contamination of cfDNA in the medium (10–12), which lowers the accuracy of NICS detection. Previous research has demonstrated better NICS results by altering the collection method of culture media in NICS. For instance, Xu et al. (13) reported a 100% successful amplification rate of chromosomes in NICS by collecting culture media (from day 3 to day 5) from thawed day 3 frozen embryos cultured to the blastocyst stage. Further, Rubio et al. (14) After washing the embryos and performing NICS, it was found that the overall ploidy consistency of the embryos was 78.7%.

The aim of this study was to explore a new method of sample collection in the culture media to reduce the impact of genetic composition on cfDNA and decrease non-embryonic cfDNA contamination.

## 2 Materials and methods

### 2.1 Research subjects

We collected and analyzed 68 blastocysts (12 couples) from patients who underwent PGT at the Hubei Maternal and Child Health Hospital Reproductive Medicine Center from February 2021 to September 2022. This study was approved by the Medical Ethics Committee of Hubei Maternal and Child Health Hospital (Ethics Approval Number [2021]: IEC (xm036) No.), and all participating couples signed informed consent forms after being fully informed about the study.

### 2.2 Sample collection

In this study, ICSI was used for fertilization in all PGT-assisted pregnancies. After successful fertilization, Day 3 post-fertilization embryos developed from zygote with two pronuclei were transferred into blastocyst culture medium for single droplet culture (embryo culture conditions: 37°C, 24 h culture duration, embryo culture medium: Quinn’s Advantage Blastocyst Medium, ooperSurgical, Trumbull, CT,USA). The embryos were cultured until Day 4 and subsequently washed. To avoid contamination, each embryo was transferred using a suction tube with a diameter of 170 µm into a new droplet of blastocyst culture medium for washing. The embryos were randomly divided into two groups based on the number of washings, namely the NICS1 group with three washings (embryos were placed in a new blastocyst culture medium droplet dish, washing was performed by the addition and subsequent aspiration of culture medium microdroplets, and the washing process was performed thrice) and the NICS2 group with ten washings (embryos were placed in a new blastocyst culture medium droplet dish, washing was performed by the addition and subsequent aspiration of culture medium microdroplets, and the washing process was performed ten times). After washing, the embryos were transferred into fresh blastocyst culture medium (25 µL per droplet per embryo) for single droplet culture continuation. Culture medium without embryos was used as the blank control. The embryos were cultured up to the blastocyst stage, and the resulting blastocysts were graded using the Gardner grading system (15), which evaluates blastocysts based on expansion status, inner cell mass, and trophectoderm development. Biopsy was performed on blastocysts with grades ≥4BB. During blastocyst biopsy, create a laser hole as far away from the inner cell mass as possible on the zona pellucida of the blastocyst, to allow the fluid in the blastocoel to drain. After the blastocyst completely collapses, use a pipette to draw 5 microliters of the culture medium mixed with blastocoel fluid and transfer it to a PCR tube, centrifuge and freeze for storage. Then, place the blastocyst in the culture dish and continue to culture for 1 hour (at 37°C) to allow the blastocyst to re-expand. The blastocysts were immobilized using a microscopic holding pipette to ensure that the inner cell mass was at the nine o’clock position, and laser drilling of the zona pellucida was performed at the three o’clock position, distant from the inner cell mass. Approximately 5–10 trophectoderm cells were obtained from the drilling site using a blastocyst biopsy needle, and the trophectoderm cells in the biopsy needle were separated from the blastocysts by micro laser combined with mechanical cutting. Biopsied blastocysts were cultured in individual culture dishes and subsequently frozen. The 5–10 trophectoderm cells obtained earlier were transferred into an PCR tube, centrifuged for 30 s to allow settling of the cells at the bottom of the tube, and stored at −80°C until further analysis.

### 2.3 Whole genome amplification and high-throughput sequencing

After the lysis of the culture medium sample, based on NICSInst whole genome amplification technology, the NICS amplification concentration was ≥0.5 after two rounds of PCR amplification. Then, the library was purified, constructed, sequenced on the machine, and combined with high-throughput sequencing, and non-invasive screening of chromosome aneuploidy was performed on the blastocyst culture medium sample. TE biopsy uses the “MALBAC Lab Single Cell Library Building Technology” based on MALBAC (multiple annexing and looping-based amplification cycles) technology for whole genome amplification and library building, combined with high-throughput sequencing (Suzhou, China).

### 2.4 Data analysis

Data processing and statistical analyses were performed using the SPSS25 software. The amplification success rate and karyotype concordance rate are expressed as percentage (%). The comparison of concordance rates between the NICS1, NICS2, and TE groups was performed using the chi-square test. P<0.05 was considered statistically significant. Due to the significant resolution difference between NICS and biopsy, this study adopted a 10 M resolution and reported chromosomal abnormalities with a fusion rate (chimerism) of over 50% in the NICS results, following the clinical transplantation process. Karyotype (chromosomal) concordance was defined as follows: 1) Complete concordance, where the chromosomal status of all chromosomes in both samples is exactly the same; 2) Partial concordance, when both NICS and PGT methods detect the same chromosomal abnormality (regardless of type: aneuploidy, copy number variation or fusion). When both NICS and PGT results indicated either chromosomally normal or abnormal, the chromosomal results were considered concordant. For evaluation of the diagnostic efficacy and effectiveness, the rates (%) of concordance between karyotype and gender, as well as diagnostic effectiveness and diagnostic efficacy indicators, including false-positive (misdiagnosis) rate, false-negative (missed diagnosis) rate, sensitivity, specificity, positive predictive value, negative predictive value, positive likelihood ratio, and negative likelihood ratio, were determined.

## 3 Results

### 3.1 Results of successful amplification of TE and NICS

This study included a total of 68 blastocysts, and all of them showed successful amplification in TE biopsies with a success rate of 100% (68/68), as shown by TE biopsy data. After different numbers of embryo washings before blastocyst formation, the NICS1 group had a successful amplification rate of 76.7%(23/30), and the NICS2 group had a successful amplification rate of 89.5% (34/38). However, the difference in the success rate between the NICS1 and NICS2 groups was not statistically significant (P>0.05) (**Table 1**).

**Table 1:** Amplification of cfDNA in embryo culture medium.

	NICS1 group	NICS2 group	*P value*
Success rate	76.7% (23/30)	89.5% (34/38)	0.15

To further clarify the influence of the number of days of embryo culture on the successful amplification rate, we found that there were 44 samples from embryos cultured until day 5, with a successful amplification rate of 75.0% (33/44), and 24 samples from embryos cultured until day 6, with a 100% successful amplification rate (24/24). The difference in successful amplification rates between the embryo culture medium samples on the 5th and 6th day was statistically significant (P<0.05) (**Table 2**). No cfDNA was detected in the samples of the blank control group. Differences in the proportions of Day 5 and Day 6 embryo culture medium samples between the NICS1 and NICS2 groups were not statistically significant (P>0.05) (**Table 3**).

**Table 2:** Amplification of cfDNA in culture medium of embryos from different days of culture.

	Day 5 medium	Day 6 medium	*P value*
Success rate	75.0% (33/44)	100.0% (24/24)	0.006

**Table 3:** Proportions of Day 5 and Day 6 blastocysts in the NICS1 and NICS2 groups.

	Day 5 blastocyst	Day 6 blastocyst	*P value*
NICS1	70.0% (21/30)	30.0% (9/30)	0.417
NICS2	60.5% (23/38)	39.5% (15/38)	

### 3.2 Chromosomal concordance of TE and NICS in the study

To further study the accuracy of NICS testing, we used TE as the gold standard. Among the embryo culture media samples with successful amplification in NICS, the chromosomal concordance rate was 43.5% (10/23) in the NICS1 group and 73.5% (25/34) in the NICS2 group. A significant difference was noted in the chromosomal karyotype concordance rate between the NICS1 and NICS2 groups (P=0.02) (**Table 4**). The chromosomal karyotype results from the NICS1 group and TE biopsies are shown in **Table 5-1**, and the chromosomal karyotype results from the NICS2 group and TE biopsies are shown in **Table 5-2**. Differences in the karyotype concordance rates of D5 and D6 embryos between the NICS1 and NICS2 groups were not statistically significant (P>0.05, **Tables 6-1**, **6-2**).

**Table 4:** Chromosome karyotype concordance by NICS.

	NICS1 group	NICS2 group	*P value*
Karyotype concordance rate	43.5%(10/23)	73.5% (25/34)	0.02

Table 5-1Karyotype results of the NICS1 group by NICS and TE.

**Table:** 

Number	Blastocyst days	NICS test results	PGT test results
A#1	5	46,XN	46,XN,-3p
A#2	6	**46,XN,-3p**	**46,XN,-3p**
B#1	5	**46,XN**	**46,XN**
B#2	5	50,XN,+Xq,+1p,+1q,+2q,+3p,+3q,+4p,+4q,-6q,+9q,+10,-14q,+15q,+19p,+20	46,XN
B#3	5	46,XN	47,XN,+10
C#1	5	45,XN,+Yq,+5q,+6p,+6q,-8p,-8q,-11p,-11q,-12,-14q,+15q, +17p,+17q,+21	46,XN
C#2	5	46,XN,-1,+2p,+2q,-4,-12,+13,+15,+18,+21,+22	46,XN
C#3	6	46,XN,+Xq,+2p,+2q,+3p,+3q,+4p,+18p,+18q	46,XN
D#1	5	45,Y,-X	46,XN
D#2	5	N/A	46,XN
D#3	6	**46,XN,-1p,-1q,+2q,+21**	**46,XN,-2q**
D#4	6	46,XN,+11q	46,XN
E#1	5	46,XN,+Xp,+19p,+19q	46,XN
E#2	5	N/A	45,X,-X(×1),+10p,+10q
E#3	5	N/A	46,XY,+Xp,+Xq
E#4	5	N/A	46,XN
E#5	5	46,XN,+9q,+11p,+18p	45,X,-X
E#6	6	**47,XXY,+X,+4p,+4q**	**48,XXY,+X,+4,-22q**
F#1	5	**46,XN,+Xp,+Xq,-Y,-18p,-18q**	**45,XN,+15q,-18p,-18q**
F#2	6	46,XN,+14q	46,XN
F#3	6	**46,XN,+X,+5p,+5q,-18p**	**46,XN,+5p,+5q,-18p,-18q**
F#4	6	46,XN	45,XN,+9p,-18p,-18q
G#1	5	N/A	46,XN
G#2	5	N/A	46,XN
G#3	5	**46,XN**	**46,XN**
G#4	5	**46,XN**	**46,XN**
G#5	5	**46,XN**	**46,XN**
G#6	5	N/A	46,XN
G#7	5	**49,XY,+Xq,+1,+11,+15**	**45,XN,-15**
G#8	6	46,XY,+3p,+4p,+4q,-5p,+5q,+6p,+9p,+12q,+17p,+18p,+18q	46,XN

**Table 5-2:** Karyotype results of the NICS 2 group by NICS and TE.

Number	Blastocyst days	NICS test results	PGT test results
H#1	5	46,XN,+Xp,+Xq,-Yp,-Yq	46,XN
H#2	5	**46,XN**	**46,XN**
H#3	5	46,XN,+X,-Y	46,XN
H#4	5	46,XN	47,XN,-9,+14
H#5	5	**46,XX**	**46,XN**
H#6	5	46,XN	45,XN,-14
H#7	5	**46,XN**	**46,XN**
H#8	6	**45,XN,-14,+18p,+20p,+21**	**46,XN,-14,+21**
H#9	6	**46,XN,-13**	**45,XN,-13**
H#10	6	**45,XN,-13**	**45,XN,-13**
I#1	5	**46,XN,-22**	**45,XN,-22**
I#2	5	46,XN,+1p	46,XN
I#3	5	N/A	46,XN,-11q,-22q
I#4	6	**46,XN,-11p,-11q,+22**	**47,XN,-11q,+22**
I#5	6	**45,XN,-9,-17q**	**46,XN,-9p,+9q**
I#6	6	**46,XN**	**46,XN**
I#7	6	**46,XN,+11q,-22q**	**45,XN,-Xq,-7q,+11q,-22**
I#8	6	**46,XN**	**46,XN**
I#9	6	**46,XN,-11q,+22**	**46,XN,-11q,+22**
J#1	5	N/A	46,XN
J#2	6	47,XN,+1p,-1q,-2p,-2q,+3p,-5q,+12,+14q,+17q,-19p,-19q,-21,+22	46,XN
J#3	5	**46,XN**	**46,XN**
J#4	6	**46,XN,+16p,+16q**	**46,XN,+16p,+16q**
K#1	5	48,XY,+Yp,-1p,+2p,+2q,-3q,-5p,-5q,-8p,+9,-10q,+11q,+12p,+12q,+12q,-16p,+18,-19,-21,+22	46,XN
K#2	5	**46,XN**	**46,XN**
K#3	5	**46,XN**	**46,XN**
K#4	5	**46,XN**	**46,XN**
K#5	5	N/A	46,XN
K#6	5	**46,XN**	**46,XN**
K#7	5	**46,XN**	**46,XN**
K#8	6	46,XN,-6,-19	46,XN
K#9	6	**46,XN**	**46,XN**
M#1	5	**49,XN,+18,+19q,+20**	**46,XN,-14q,+20p,+20q**
M#2	5	**44,XN,-18,-21**	**44,XN,-18,-21**
M#3	5	N/A	45,XN,-15
M#4	5	**47,XN,-3,+5q,+15q,+16,-21**	**48,XN,+15,+16p,+16q,+19**
M#5	6	**46,XY,-11p**	**46,XN,-11p**
M#6	6	46,XN	46,XN,-2p

N/A means no result. The chromosome results in bold indicate a consistent karyotype in the table. The number represents the embryo number, and the same letter indicates that it originated from the same couple.

Table 6-1Karyotype concordance rates of blastocysts with different durations of culture in the NICS1 group.

**Table:** 

	Day 5	Day 6	*P value*
Karyotype concordance rate	42.8% (6/14)	44.4% (4/9)	1.00

**Table 6-2:** Karyotype concordance rates of blastocysts with different durations of culture in the NICS2 group.

	Day 5	Day 6	P value
Karyotype concordance rate	68.4% (13/19)	80.0% (12/15)	0.69

### 3.3 Detection and diagnosis efficiency of NICS

To verify the reliability of NICS for detecting chromosomal abnormalities in embryos, we compared the diagnostic efficiency of detecting chromosomal abnormalities between the NICS1 and NICS2 groups using TE as the gold standard (**Table 7**). The sensitivity and specificity of detecting chromosomal abnormalities were better in the NICS2 group than in the NICS1 group (83.3% vs 60.0%; 62.5% vs 30.8%, respectively). The false-positive (misdiagnosis) rate and false-negative (missed diagnosis) rate were lower in the NICS2 group than in the NICS1 group (37.5% vs 69.2%; 16.7% vs 40.0%, respectively).

**Table 7:** Diagnostic efficiency of NICS in detecting chromosomal abnormalities.

Chromosomal detection results of NICS with TE as the gold standard		
Evaluation index	NICS1 group (n=23)	NICS2 group (n=34)
SE	60.0%	83.3%
SP	30.8%	62.5%
FPR	69.2%	37.5%
FNR	40.0%	16.7%
PPV	40.0%	71.4%
NPV	50.0%	76.9%
PLR	0.9	2.2
NLR	1.3	0.3

Sensitivity (SE) = (true positive)/(true positive + true negative); specificity (SP) = (true negative)/(true negative + false positive); false-positive rate (FPR) = (false positive)/(true negative + false positive); false-negative rate (FNR) = (false negative)/(true positive + false negative); positive predictive value (PPV) = (true positive)/(true positive + false positive); negative predictive value (NPV) = (true negative)/(true negative + false negative); positive likelihood ratio (PLR) = sensitivity/false-positive rate: negative likelihood ratio (NLR) = false-negative rate/specificity.

## 4 Discussion

In this study, we compared the impact of washing embryos three times and ten times before transferring them to fresh culture media for continued development until the blastocyst stage in NICS. The results suggest that washing embryos ten times before NICS testing provides certain advantages. By optimizing the NICS sample collection method, cfDNA (genetic material) in the medium was extracted. This study offers a novel solution to reduce non-embryonic cfDNA contamination in embryo culture media, presenting an optimized sampling approach for future clinical applications of NICS testing.

In current studies, the concordance rate between the chromosome from culture medium and embryo TE chromosome ranges from 30% to 84% (12, 16, 17), and different research institutions yield different results. According to this study, the karyotype concordance rate between NICS results and TE results in the NICS sampling method reached 73.5% after the embryos were washed ten times, and it was noticeably higher than those washed three times, which increased the accuracy of NICS detection. However, we are also considering if more frequent washings and the ensuing longer exposure times for the embryos could have an effect on formation.

In the study, despite laser drilling collapsing the blastocyst and releasing the cfDNA in the blastocyst fluid into the medium, 76.7% and 89.5% of the medium samples in the NICS1 group and NICS2 group, respectively, were effectively amplified. The amplification rate efficiency is less than that of those reported in earlier investigations (17, 18). After washing the embryos and switching the culture medium for the embryos, there may be a decrease in the concentration of cfDNA in the medium. The effective amplification rates of the two groups did not differ substantially, proving that the number of washing cycles had no impact on the amount of cfDNA present in the embryo culture media used for WGA. The success rate of cfDNA amplification in the embryo culture medium on day 6 in this study was higher than that on day 5 (100% vs. 75.0%), which is consistent with previous studies regarding the relationship between the concentration of cfDNA in the culture medium and the length of the embryo culture time (19).

In a recent study, Capalbo et al. (20) proposed that transfer of low mosaic embryos (<50%) is feasible and has comparable embryonic developmental potential and neonatal outcomes as those of transfer of euploid embryos. In this study, a resolution of 10 M/whole arm and >50% chimerism were reported as chromosomal abnormalities, as per the criteria from the clinical transplantation process. Using TE biopsy as the gold standard, it was found that the diagnosis of chromosomal abnormalities was more favorable in the NICS2 group. The study has some limitations, including the small number of blastocysts available. However, the TE results were used as the gold standard. Although it complied with the clinical operation procedures, some research revealed that TE biopsy could not accurately reflect the embryo’s chromosomal state (21), and false negatives may occur.

In this study, embryos were washed ten or three times, and there was no difference in the chromosome amplification rate between the two groups of culture media. After ten cycles of embryo washing, the consistency rate of the NICS chromosome karyotype was higher. The research results provide a new solution for reducing cfDNA contamination from non-embryonic sources in embryo culture media. In the future, it is necessary to further reduce the gradient of washing times, find more suitable washing times, optimize the sampling method of embryo culture medium for NICS detection, reduce the level of cfDNA contamination from non-embryonic sources in the culture medium, improve the accuracy of NICS detection, and enable NICS technology to more accurately evaluate the chromosome status of embryos, select high-quality embryos, and improve the success rate of assisted reproductive technology.

## Data availability statement

The original contributions presented in the study are included in the article/supplementary material, further inquiries can be directed to the corresponding author.

## Ethics statement

The studies involving humans were approved by Medical Ethics Committee of Hubei Maternal and Child Health Hospital. The studies were conducted in accordance with the local legislation and institutional requirements. The participants provided their written informed consent to participate in this study.

## Author contributions

XK: Writing – original draft. MW: Writing – original draft. HS: Writing – review & editing, Supervision, Funding acquisition. JZ: Writing – review & editing. FP: Writing – review & editing. NZ: Writing – review & editing. ZC: Writing – review & editing. YW: Writing – review & editing.
